# Dietary Taurine Regulation of the Intestinal Microbiome in Chinese Stripe-Necked Turtle (*Mauremys sinensis*)

**DOI:** 10.3390/ijms26020445

**Published:** 2025-01-07

**Authors:** Yue Yuan, Xin Niu, Chenguang Hao, Lingyue Liang, Zubin Huang, Dongmei Wang, Meiling Hong, Li Ding

**Affiliations:** 1 Key Laboratory of Tropical Animal and Plant Ecology of Hainan Province, Ministry of Education, Key Laboratory for Ecology of Tropical Islands, College of Life Sciences, Hainan Normal University, Haikou 571158, China; 202312071300003@hainnu.edu.cn (Y.Y.); 202311071300001@hainnu.edu.cn (X.N.); lianglingyue199613@163.com (L.L.); huang113013@163.com (Z.H.); 2 Institute of Tropical Bioscience and Biotechnology, Chinese Academy of Tropical Agricultural Sciences, Haikou 571158, China; haochenguang0121@catas.cn (C.H.); wdmei1969@126.com (D.W.)

**Keywords:** *Mauremys sinensis*, gut microbiota, animal health, nutrient additives

## Abstract

Taurine is essential for sustaining the body’s physiological equilibrium and is extensively utilized as a dietary supplement and immune system enhancer for aquatic creatures. The gut microbiota serves as a vital health indicator in animals. In this study, our goal was to evaluate the effects of dietary taurine on the gut microbiome of Chinese stripe-necked turtles (*Mauremys sinensis*). Turtles were evenly divided into three supplementation groups: a control group with no taurine in the diet, a low group with 0.1% taurine, and a high group with 0.4% taurine. High-throughput sequencing was employed to analyze the gut microbiome’s composition and structure. The results showed that the impact of taurine supplementation on the α-diversity and β-diversity of the gut microbiome was not statistically significant. At the phylum level, Firmicutes and Bacteroidetes predominated the gut microbiome. At the genus level, three beneficial bacteria, *Clostridium_sensu_stricto_13*, *Pygmaiobacter*, and *Terrisporobacter* showed significant differences under different levels of taurine supplementation and increased in abundance with the supplementation of taurine, while the harmful bacterium *Brucella* decreased in abundance with the addition of taurine and exhibited significant differences. Furthermore, LEfSe analysis and functional predictions highlighted significant variations in the functional traits of gram-positive intestinal bacteria among the different taurine supplementation levels. These findings imply that dietary taurine may alter the intestinal microbiome structure in Chinese stripe-necked turtles, offering valuable insights for the scientific assessment of taurine’s health benefits as a feed additive and potentially guiding the formulation of more informed and healthier feeding practices.

## 1. Introduction

Taurine, a sulfur-containing amino acid with a straightforward structure, is the most abundant in living organisms and is predominantly found in animal tissues and cells that make up the nervous, muscular, and glandular systems. In a previous study, the uptake and distribution of taurine in turtle bladder epithelial cells was described in detail [1]. They play a vital role in maintaining normal vision, regulating nerve impulses, participating in bodily endocrine functions, and enhancing immune system capabilities [2]. In a study on anoxia-tolerant painted turtles, it was found that under anoxia, taurine levels increase about 24 times, suggesting that taurine may play a role in regulating the electrical activity of the anoxic turtle brain [3]. The contemporary aquaculture sector strives for excellence and productivity, but high-density farming practices can weaken fish stress resistance, increase disease incidence, and lower survival rates. As a result, taurine has emerged as a prevalent feed additive and immune booster due to its unique benefits [4]. Research by Wei et al. [5] demonstrates that supplementing the diets of whelk (*Pseudorasbora parva*) and loach (*Oriental weatherfish*) with varying levels of taurine improved their hypoxia tolerance, thereby enhancing their ability to withstand low oxygen conditions. Gwang-Sic Park’s study indicated that a diet containing 1.4% taurine significantly influenced the growth and development of flounder (*Paralichthys olivaceus*), promoting growth by increasing protein efficiency but inhibiting it at higher taurine concentrations [6]. Moreover, taurine bolsters animal immunity; Liang’s study found that taurine supplementation at both low and high concentrations to rats of different ages (*Muroidea*) significantly reduced the immunosuppressive effects in young rats and alleviated the degenerative impacts on some organs in older rats [7]. Hou’s experiment also demonstrated that the addition of taurine in feed with low fishmeal levels can improve the growth performance and antioxidant capacity of the Chinese softshell turtle [8]. Taurine also acts as a key signaling molecule in the gut, positively impacting intestinal instability, and its supplementation has been shown to significantly regulate intestinal flora and strengthen intestinal immunity against antibiotic exposure [9].

The intestinal tract of animals hosts a vast community of gut microbes, which rely on the host’s intestines for survival and, in return, assist the host in performing numerous physiological and biochemical functions [10]. These gut microorganisms significantly influence the host’s health, not only by participating in metabolic processes but also by being intricately linked to the operation of several systems, including the digestive, immune, and neurological systems [10,11,12,13]. The microbial communities are crucial for the intestinal ecosystems of reptiles [14,15], humans [16], and various other mammals [17,18].

The Chinese stripe-necked turtle (*Mauremys sinensis*), alternatively known as the Chinese flower turtle or grass turtle, is a species that is predominantly aquatic in nature [19]. This turtle species is widely found in Laos, Vietnam, and throughout China, being one of the most extensively distributed freshwater turtles within the country. Its wild populations are currently classified as critically endangered. In China, it is predominantly located along the coastal regions of Zhejiang and South China, and it primarily consumes green grasses and other aquatic vegetation, indicating its omnivorous diet [19]. Over recent years, efforts to bolster the population of the Chinese stripe-necked turtle have led to a significant expansion in its breeding programs. While this has fostered the growth of turtle farming, it has also introduced challenges such as the degradation of the farming environment, a decline in turtle immunity, and an increase in disease incidence [20]. Consequently, there is an urgent need to address the enhancement of turtle immunity and the safeguarding and management of illnesses among these creatures [21].

In this study, we compared the makeup and organization of gut microorganisms in Chinese stripe-necked turtles (*Mauremys sinensis*) by adding different concentrations of taurine to diets, aiming to reveal the systematic effects of taurine supplementation on the diversity and function of gut microorganisms. This study will provide a scientific rationale for employing taurine as a feed additive in the breeding of turtle species and animal farming.

## 2. Results

### 2.1. Analysis of rRNA Sequencing Results

The number of quality-filtered sequences measured for each sample was 31,146–63,641, totaling 839,095 sequences. The length distribution was 404–419 bp, with an average length of 408 bp (Supplementary File). All samples sequenced were sparse, with 30,313 reads, and all sparse curves attained the plateau phase, signifying that an adequate sampling depth had been reached for every sample.

### 2.2. Colony Composition and Relative Abundance

**Figure 1:**
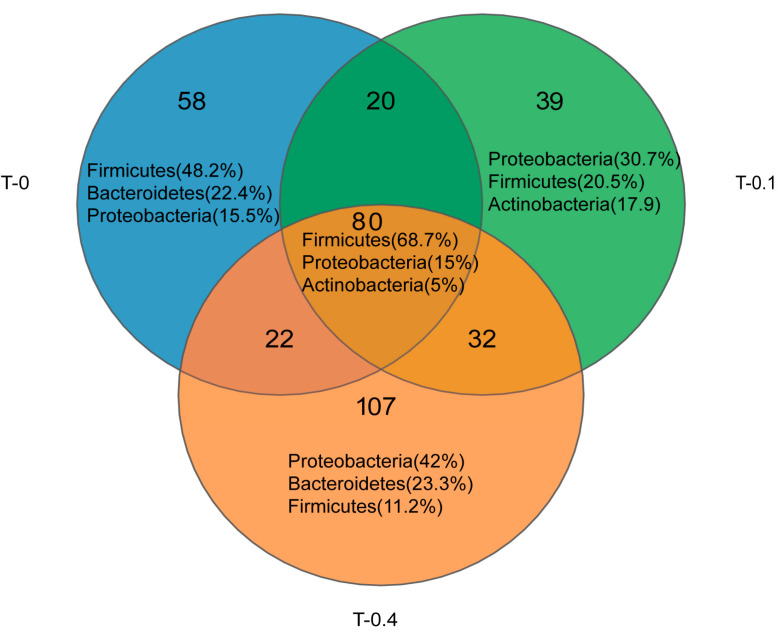
Venn diagram illustrating the unique and shared gut flora of *Mauremys sinensis* OTU at different taurine supplementation levels.

Venn diagrams were employed to illustrate the commonalities and disparities in the gut microbiota of juvenile Chinese stripe-necked turtles across different groups with varying taurine supplementation (a control group without taurine, a 0.1% taurine group, and a 0.4% taurine group). Out of a total of 358 operational taxonomic units (OTUs) identified in the gut microbiota, 80 OTUs (22.35%) were found to be common across all groups. Within these shared OTUs, 55 belonged to the phylum Firmicutes (representing 68.7% of the shared OTUs), 12 to the phylum Proteobacteria (15% of the shared), and four to the phylum Actinobacteria (5% of the shared). In the control group, 58 OTUs (16.2% of the total) were unique, with 28 OTUs (48.2% of the unique) affiliated with Firmicutes, 13 OTUs (22.4% of the unique) with Bacteroidetes, and nine OTUs (15.5% of the unique) with Proteobacteria. For the low group, 39 OTUs (10.89% of the total) were unique to the Chinese stripe-necked turtles, consisting of 12 OTUs (30.7% of the unique) with Proteobacteria, and eight OTUs each with Firmicutes and Actinobacteria (20.5% and 17.9% of the unique, respectively). In the high group, 107 OTUs (29.89% of the total) were unique, including 45 OTUs (42% of the unique) with Proteobacteria, 25 OTUs (23.3% of the unique) with Bacteroidetes, and 12 OTUs (11.2% of the unique) with Firmicutes (Figure 1).

**Figure 2:**
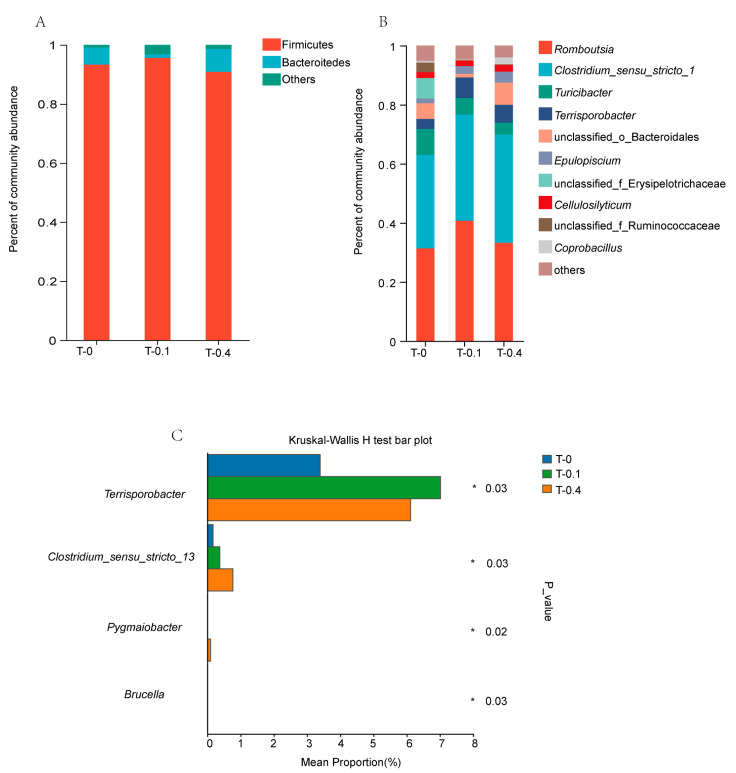
Composition and differences of gut microbiota in *Mauremys sinensis* under different treatments. (**A**) represents the relative abundance of intestinal microbiota at the phylum level; (**B**) represents the relative abundance of intestinal microbiota at the genus level; (**C**) represents the differences in intestinal microbiota at the genus level. *p* < 0.05 reflects a statistically significant difference, with significant intergroup differences denoted by *.

At the phylum level, the dominant phylum (>1%) in the three groups were Firmicutes, Bacteroidetes, and the remaining other phyla (Figure 2A). The phylum Firmicutes accounted for 93.34%, 95.62%, and 90.89% of the three groups of samples, and Bacteroidetes accounted for 5.78%, 1.21%, and 7.73% of the three groups of samples, respectively. The proportions of the remaining other phyla were 0.88%, 3.17%, and 1.37%, respectively.

At the genus level, the genera with higher relative abundance in the three groups were *Romboutsia*, *Clostridium_sensu_stricto_1*, *Turicibacter,* and *Terrisporobacter* (Figure 2B). In the control group, the percentage of *Romboutsia* was 31.35%, *Clostridium_sensu_stricto_1* 31.67%, *Turicibacter* 8.75%, and *Terrisporobacter* 3.39%. In the low group, the percentage was 40.69% for *Romboutsia*, 35.97% for *Clostridium_sensu_stricto_1*, 5.59% for *Turicibacter*, and 7.02% for *Terrisporobacter*. In the high group, the proportion of *Romboutsia* was 33.22%, that of *Clostridium_sensu_stricto_1* was 36.62%, that of *Turicibacter* was 4.03%, and that of *Terrisporobacter* was 6.11%. The relative abundance of *Clostridium_sensu_stricto_13* and *Pygmaiobacter* in the high group was significantly higher than that in the control and low groups (*p* = 0.03 and *p* = 0.02, Figure 2C). The relative abundance of *Terrisporobacter* was significantly higher in the low group than in the control and high groups (*p* = 0.03, Figure 2C). The comparative prevalence of *Brucella* was markedly greater in the control group compared to that in both the low and high groups (*p* = 0.03, Figure 2C).

**Figure 3:**
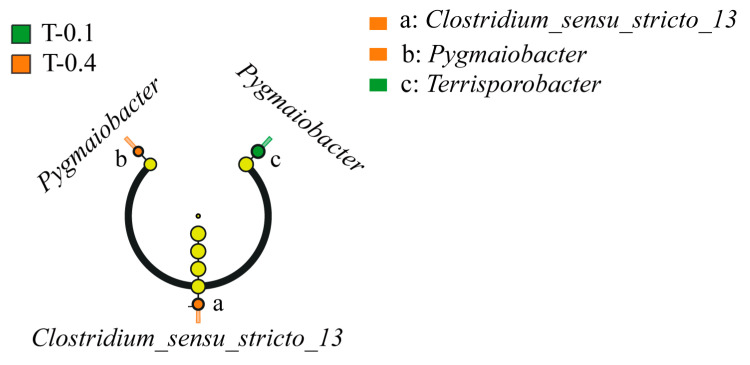
LEfSe analysis was conducted on the intestinal microbiota biomarkers of *Mauremys sinensis* under different treatments. The diameter of each circle reflects its abundance. The multi-class analysis is flexible, requiring at least one class difference. The circles from the inside out reflect the taxonomy from phylum to genus. Alternatively, the circles from the inside out symbolize the taxonomic classification from phylum to genus. Class, order, and family labels will be displayed. All taxa with an LDA score > 2 will be shown.

The intestinal flora of the turtles treated with different levels of supplementation were analyzed by LDA (LDA > 2), and the taxonomic units of the differences in the intestinal flora of the turtles in the different groups were counted. From the phylum level to the genus level, a total of three differential features were found, two of which were in the high group and one in the low group, and the intestinal microorganisms of the Chinese stripe-necked turtles in the low group were significantly enriched with the genus *Terrisporobacter*, and the Chinese stripe-necked turtles in the high group were enriched with the genera *Clostridium_sensu_stricto_13* and *Pygmaiobacter* (Figure 3).

### 2.3. Alpha and Beta Diversity Analyses

**Figure 4:**
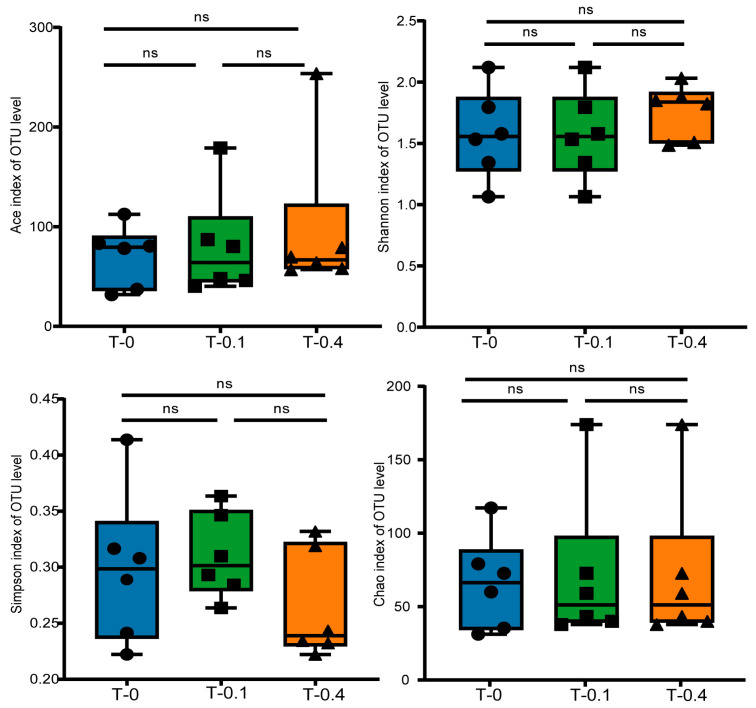
Alpha diversity of intestinal microbiota in *Mauremys sinensis* under different taurine supplementation levels. Alpha diversity of bacteria measured by Ace, Shannon, Simpson, and Chao was tested for significance using the Kruskal–Wallis rank-sum test, with *p*-values indicating the confidence level of the statistical analyses; *p* < 0.05 reflects statistically significant differences. ns indicates nonsignificant differences.

The alpha diversity indices of the intestinal bacterial communities of the turtles under different treatments (control, low, and high groups) are shown in Figure 4. The Ace, Shannon, Simpson, and Chao indices of the intestinal bacterial communities were not significantly different among the different taurine treatments.

**Figure 5:**
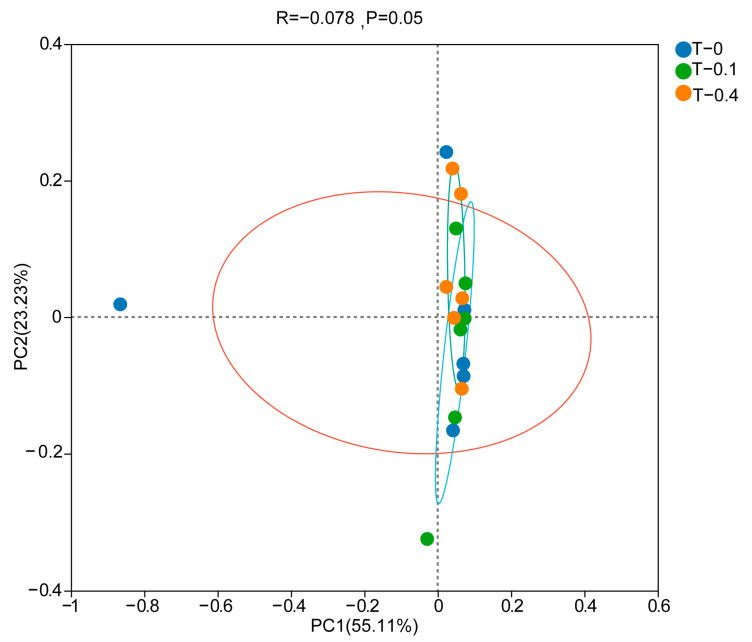
Beta diversity of intestinal microbiota. PCoA analysis diagram of beta diversity based on weighted UniFrac distances in *Mauremys sinensis* under different treatments. The vertical axis represents the principal coordinate component that explains the maximum possible variation in the data, and the horizontal axis represents the principal coordinate component that accounts for the largest proportion of the remaining variation after the vertical axis has explained the maximum variance. The R value represents the goodness of fit of the model; the higher the R^2^ value, the stronger the model’s explanatory power, and the better it reflects the differences and similarities among samples. The *p*-value indicates the confidence level of the statistical analysis; *p* < 0.05 reflects a statistically significant difference. Colony composition and relative abundance.

Beta diversity analyses showed that the intestinal flora did not differ significantly among taurine supplementation conditions (Figure 5).

### 2.4. Predicted Functional Analysis

**Figure 6:**
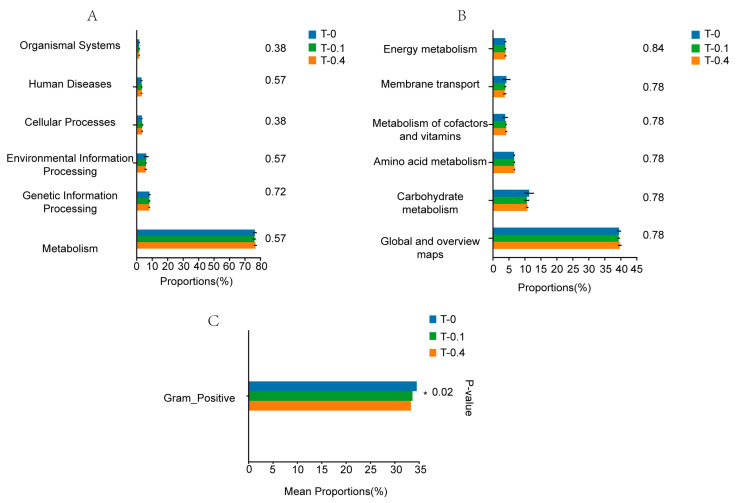
BugBase phenotype prediction of gut microbiota in *Mauremys sinensis* in different taurine supplementation groups. Functional analysis of bacteria was conducted using PICRUSt2; bacterial phenotypes were identified using the BugBase method. (**A**) shows that the relative abundance of predicted bacterial genes associated with level 1 KEGG pathways significantly varies across the macroscopic genome. (**B**) shows the abundance of level 2 KEGG pathways and functional pathways. To determine functional and phenotypic differences in *Mauremys sinensis* fed different doses of taurine, bacterial phenotypes were analyzed and predicted using the BugBase algorithm. (**C**) shows that based on a one-way ANOVA of phenotypic differences between groups, the vertical axis represents the phenotype name, and the horizontal axis represents the percentage value of the relative abundance of a certain phenotype in the sample. *p* < 0.05 reflects a statistically significant difference, with significant intergroup differences denoted by *.

The three types of Chinese stripe-necked turtles’ gut microbiota mostly carried out tasks pertaining to metabolism, cellular processes, human diseases, genetic information processing, environmental information processing, and organismal systems. (Figure 6A). These include extensive and worldwide routes such as the metabolism of carbohydrates, amino acids, cofactors, and vitamins and membrane transport and energy metabolism. However, these metabolic pathways did not significantly differ across the three groups (Figure 6B).

To obtain a deeper understanding of the variations in the intestinal bacterial flora of Chinese stripe-necked turtles across the three groups, the BugBase algorithm was utilized to predict the bacterial phenotypes. The results indicate that gram-positive bacteria were significantly different among the three groups (Figure 6C), with notable variations observed in the gram-positive bacterial category (Figure 6C).

## 3. Discussion

The animal gut microbiota is a complex and fine-grained ecosystem. These microorganisms play a crucial role in digestion, immunity, physiology, and behavior, and an analysis of gut microbiota function can help us to understand the ecological adaptations of the host [22]. In this study, we thoroughly examined the effects of taurine supplementation on the intestinal microbial population of the Chinese stripe-necked turtle (*Mauremys sinensis*) using high-throughput Illumina sequencing technology. The results showed that the intestinal microbial composition of the Chinese stripe-necked turtle (*Mauremys sinensis*) changed with different concentrations of taurine.

In this study, we observed no substantial variations in the diversity of the gut microbiota in Chinese stripe-necked turtles across varying concentrations of taurine. Similarly, a β-diversity analysis revealed no significant differences among the three groups. This lack of variation may be attributed to the turtles already containing a certain level of taurine in their bodies, meaning that additional taurine in their diet did not lead to notable changes in their intestinal microbial communities [23]. In the gut microbiome, *Terrisporobacter* bacteria is associated with the health of the host, and a higher abundance is beneficial to the survival of the host. They produce various short-chain fatty acids (SCFAs) in the carbohydrate metabolism pathway, which are beneficial to gut health [24]. *Terrisporobacter* is associated with the host’s health, with a higher abundance being beneficial for the host’s survival, and may play an important role in anti-inflammatory processes [25]. It can also help the host survive in harsh environments [24]. However, the abundance of *Terrisporobacter* in the intestinal tract of the turtles varied significantly with different levels of taurine supplementation, peaking at low taurine concentrations and decreasing with higher supplementation. This suggests that taurine supplementation may play a role in regulating the balance of intestinal microorganisms, potentially aiding in the maintenance of intestinal health and overall host well-being. *Clostridium_sensu_stricto_13*, a member of the gut microbial community, is known to support gut health and homeostasis. It is capable of generating short-chain fatty acids, with a particular emphasis on butyric acid, through carbohydrate fermentation, which are vital for gut health as they provide energy to intestinal epithelial cells, enhance intestinal barrier function, and interact with the immune system [26]. Studies have indicated that *Clostridium_sensu_stricto_13* can reduce inflammation by producing IL-10 and can induce the accumulation and differentiation of Treg cells, thereby alleviating colitis and allergic diarrhea in mice [27]. In this study, the abundance of *Clostridium_sensu_stricto_13* increased with higher taurine supplementation, indicating that taurine may positively influence the enhancement of intestinal barrier function and immunity in Chinese stripe-necked turtles. Additionally, the genus *Pygmaiobacter* is closely linked to host health, and fluctuations in its abundance may correlate with the development of certain diseases. They may also exhibit prebiotic properties, enhancing the proliferation of advantageous gut bacteria and bolstering the architecture and efficacy of the intestinal microbiome [28]. The abundance of *Pygmaiobacter* in the turtles’ intestines varied significantly with different taurine levels, increasing with higher taurine supplementation. This suggests that increased taurine intake may enhance the intestinal health of Chinese stripe-necked turtles and reduce their disease risk.

*Brucella*, a group of Gram-negative, non-motile bacteria, primarily infects a wide range of mammals, including humans, causing brucellosis [29]. This disease can present as subacute or chronic lesions in animals [30], and if untreated, may lead to chronic infections that adversely affect the survival and reproduction of the host [31]. In this study, the abundance of *Brucella* in the intestinal tract of Chinese stripe-necked turtles varied significantly with different taurine supplementation levels, decreasing as taurine concentrations increased. This finding further suggests that taurine supplementation may help reduce the risk of disease in these turtles.

The complex interactions between gut microbes and host physiological functions suggest that gut microbes serve as a crucial component in preserving the well-being of the host [32]. Among the multidimensional effects on host physiological functions, metabolic function is one of the most prominent and extensively researched mechanisms [33]. In this study, we found that the gut microbiota of the Chinese stripe-necked turtle exhibited diverse functional characteristics under different taurine supplementation conditions, including metabolism, genetic information processing, environmental information processing, metabolism, cellular processes, human diseases, and organismal systems. This result further demonstrates the important role of gut microbes in the metabolic activities of the Chinese stripe-necked turtle and is consistent with previous studies on gut microbes in turtles [34]. In addition, a significant difference in the gram-positive intestinal microbiota of the turtles was found under different taurine supplementation conditions, and this difference became more significant with an increase in taurine supplementation. Gram-positive bacteria may cause disease in turtles, especially if they are immunosuppressed or weakened. Although gram-positive bacteria are not generally considered to cause disease in turtles, they can cause disease under certain conditions [35]. This result suggests that changes in the gut microbiology of Chinese stripe-necked turtles may reduce the risk of disease in Chinese stripe-necked turtles when taurine is added to the diet.

## 4. Materials and Methods

### 4.1. Animals

Twenty-seven juvenile Chinese stripe-necked turtles (*Mauremys sinensis*) were acquired from Dongshan Breeding Factory in Haikou City, Hainan Province. These turtles were then housed and conditioned in a breeding room for two weeks under uniform initial rearing conditions. After acclimating to the initial environment, the juvenile turtles were categorized into three groups based on varying levels of taurine supplementation: a control group (control group: T-0) of nine turtles with no taurine in their basic diet (Table 1), a low-dose group (low group: T-0.1) of nine turtles with 0.1% taurine in a basic diet, and a high-dose group (high group: T-0.4) of nine turtles with 0.4% taurine in a basic diet. In each group, there were three duplicate tanks, with three turtles in each tank. The feeding duration for all groups was 60 days. Throughout the feeding phase, the pH level of the water ranged from 7.5 to 7.9, with the temperature fluctuating between 26 and 28 °C. The turtles received regular feeding on Mondays and Thursdays, and the water was refreshed the day following each feeding session. Following the approval of the Animal Ethics Committee of the Hainan Provincial Ecological and Environmental Education Centre (Approval No. HNECEE 2023-005), after a 60-day feeding period, two turtles were randomly selected from each tank (six turtles in each group). After dissection, their intestinal contents were collected, placed into 2 mL coded cryovials, rapidly immersed and preserved in liquid nitrogen, and subsequently stored in a −80 °C ultra-low temperature freezer for preservation.

### 4.2. DNA Extraction and PCR Amplification

Genomic DNA was isolated from all microorganisms utilizing the MagAtract PowerSoil Pro DNA Kit from Qiagen, Hilden, Germany. Agarose gel electrophoresis (1%) was used to assess the extracted genomic DNA’s integrity, and a NanoDrop 2000^®^ND-2000 was used to determine the DNA’s concentration and purity by Thermo Scientific, Wilmington, DE, USA. The V3-V4 hypervariable region of the bacterial 16S rRNA gene was targeted for amplification using an ABI GeneAmp^®^ 9700 PCR thermocycler from ABI, CA, USA. The amplification employed primers 338F (sequence 5′-ACTCCTACGGGGAGGCAGCAG-3′) and 806R (sequence 5′-GGACTACHVGGGTWTCTAAT-3′) [36]. Each sample’s PCR reaction was conducted three times in a 20 µL reaction mix comprising 10 µL of Taq Pro Multiplex DNA Polymerase, 10 ng of template DNA, 0.8 µL of each primer at a 5 μM concentration, and ddH_2_O to make up the volume to 20 µL. The PCR procedure involved a three-minute initial denaturation at 95 °C, 29 cycles (each including 30 s of denaturation at 95 °C, 30 s of annealing at 53 °C, and 30 s of extension at 72 °C), a final extension at 72 °C for 10 min, and holding at 4 °C. Thermo Fisher Scientific’s Qubit 4.0 was used to quantify the PCR products after they were recovered from a 2% agarose gel and purified using a DNA gel recovery purification kit (PCR Clean-Up Kit, Passionate, Shanghai, China). In accordance with the methods of Meggie BioMedical Technology Ltd. (Shanghai, China), the purified amplicons were combined in equimolar quantities and put through paired-end sequencing on an Illumina MiSeq PE300 platform. The raw sequencing reads were deposited in the NCBI Sequence Read Archive database (Accession Number: SRP546489).

### 4.3. Processing of Sequencing Data

The raw sequences obtained from double-ended sequencing were subjected to quality control using the fastp software [37] (https://github.com/OpenGene/fastp, version 0.19.6, accessed on 15 August 2024) and then spliced using the FLASH software [38] (http://www.cbcb.umd.edu/software/flash, version 1.2.11, accessed on 15 August 2024). The process involved the following steps: (1) The quality of the sequence tails was assessed to eliminate bases with quality scores below 20. A sliding window of 50 base pairs was applied, and if the average quality score within this window was less than 20, all bases from the starting position of the window to the end were removed. After quality control, reads shorter than 50 base pairs were filtered out, as well as those containing ambiguous ‘N’ bases. (2) Paired-end reads were merged into single sequences based on their overlap. The minimum overlap required for merging was set to 10 base pairs. (3) Within the overlap region of the combined sequences, the highest permitted error rate was 0.2. Sequences that did not meet this requirement were eliminated. (4) By locating primers and barcodes at the beginning and end of the sequences and modifying the sequence orientation appropriately, the samples were differentiated. In this case, barcodes were required to match perfectly, with zero allowable mismatches, while the primers were allowed up to two mismatches. The quality-controlled and merged sequences were then processed with UPARSE v7.1 software [39] (http://drive5.com/uparse/, accessed on 15 August 2024). A 97% similarity criteria was used to group them into Operational Taxonomic Units (OTUs), and chimeric sequences were removed. Sequences identified as chloroplasts and mitochondria were also removed from all samples. The OTUs were taxonomically classified to the species level using the RDP classifier [40] (https://ngdc.cncb.ac.cn/databasecommons/database/id/237, version 2.11, accessed on 15 August 2024) with the Silva 16S rRNA gene database (v138) as a reference. A confidence threshold of 70% was applied during this classification. Finally, the statistical analysis of species composition was conducted across diverse taxonomic levels for each sample.

### 4.4. Ecological and Statistical Analysis

Using mothur software (v.1.30.2, University of Michigan, Ann Arbor, MI, USA), sparse curves were constructed with the aim of verifying whether the sequencing depth could adequately cover the number of OTUs that would be expected under the 97% sequence similarity criterion [41]. Next, the collected datasets were analyzed for α-diversity indices using mothur to quantify the abundance and diversity of bacterial and fungal communities, and the differences between α-diversity were assessed by the Wilcoxon rank sum test. To assess the β-diversity, we used a non-metric multidimensional scaling disorder (NMDS) approach. In addition, Venn diagrams were analyzed for OTUs using the BASE and VEGAN packages in R.26. The community structure was analyzed at the taxonomic level of phylum and genus, and Bar diagrams were produced with the aid of the PANDAS package in Python (version 2.7) based on the data tables in the tax_summary_a folder [42] (where relative abundances below 1% were combined into the ‘others’ category). To explore noticeable disparities among the intestinal microbiota of Chinese stripe-necked turtles in the control group and the different taurine supplementation groups, the Kruskal–Wallis rank-sum test was used, and a *p*-value of less than 0.05 was considered to be statistically significant. A linear discriminant analysis effect size analysis of samples was also performed [36], which was based on different grouping conditions (Kruskal–Wallis *p*-value less than 0.05 and log LDA score greater than 2). In order to predict functional changes in the gut microbiota of Chinese stripe-necked turtles in the control and different taurine supplementation groups, the PICRUSt2 tool was used to analyze them. For OTU normalization, the 16S copy number was processed based on the BugBase prediction, followed by the prediction of microbial phenotypes using the pre-calculated files provided. Finally, the Kruskal–Wallis rank sum test was applied to compare the changes in the relative abundance of the gut microbes between the control group and the different taurine supplementation groups of Chinese stripe-necked turtles, and notable differences were deemed to be present only when the *p*-value was below 0.05.

## 5. Conclusions

In the present study, we explored the impact of dietary taurine supplementation on the gut microbiota. Our study uncovered alterations in the intestinal microbial composition and functionality of Chinese stripe-necked turtles with supplementation of various dietary levels of taurine. The increase in the beneficial bacteria *Terrisporobacter*, *Clostridium_sensu_stricto_13*, and *Pygmaiobacter*, the decrease in the harmful bacterium *Brucella*, and the differences in gram-positive functions indicate that the addition of taurine not only significantly modulates the gut microbiota but may also bring positive effects, such as improving gut health and enhancing immunity, which previous studies have already demonstrated. These findings provide a scientific basis for evaluating the effects of taurine as a feed additive on turtles’ health, which may help to develop healthier feeding strategies.

## Figures and Tables

**Table 1:** Basic diet composition and nutrient level (air-dried basis).

Ingredient Composition	Quantity Contained (%)
Steamed fish meal	54.00
Dehulled soybean meal	17.00
Starch	23.00
Wheat gluten	2.00
Ca(H_2_PO_4_)_2_	1.50
Choline	0.25
Multi-vitamin ^1^	0.15
Vitamin C ester	0.10
Multi-mineral ^2^	1.00
Fish oil	1.00
Total	100
Nutrient levels	
Crude protein	44.28
Crude fat	8.16
n-3PUFA	2.05
n-6 PUFA	0.65
n-3/n-6	3.18

^1^ Contained the following per kg of multi-vitamin: VA 10,000,000 IU, VB_1_ 20 mg, VB_2_ 25 mg, VB_6_ 12 mg, VB_12_ 100 mg, VD_3_ 3,750,000 IU, VE 50 IU, VK_3_ 12 mg, biotin 10 mg, folic acid 2.5 mg, D-pantothenic acid 40 mg, nicotinic acid 80 mg. ^2^ Contained the following per kg of multi-mineral: K 90 g, Mg 27 g, Cu 750 mg, Fe 13.5 mg, Mn 1.2 g, Zn 38.5 g, I 120 mg, Se 50 mg, and Co 100 mg.

## Data Availability

Data will be made available on request.

## Supplementary Materials

The following supporting information can be downloaded at: https://www.mdpi.com/article/10.3390/ijms26020445/s1.

## References

[1] Miles A.R., Hawrysh P.J., Hossein-Javaheri N., Buck L.T. (2018). Taurine Activates Glycine and GABAA Receptor Currents in Anoxia-Tolerant Painted Turtle Pyramidal Neurons. J. Exp. Biol..

[2] Li S., Ji X., Shao T., Bai Y. (2021). Research Progress on Application of Taurine in Aquaculture. Feed Res..

[3] Palmisano J., Mitchell P.P., Steinmetz P.R. (1989). NBD-Taurine Uptake by Alpha-Type Carbonic Anhydrase Cells of Turtle Bladder. Am. J. Physiol..

[4] Huang H., Shao J., Xiang L. (2005). Current Research Status and Progress of Fish Immunostimulants. J. Fish. China.

[5] Wei Z., Yu H., Wu R. (2004). Effect of Taurine on Hypoxia Resistance of *Tilapia* sp.. Amino Acids Biot. Res..

[6] Park G.-S., Takeuchi T., Yokoyama M., Seikai T. (2002). Optimal Dietary Taurine Level for Growth of Juvenile Japanese Flounder *Paralichthys olivaceus*. Fish. Sci..

[7] Liang X., Ye X., Kong Y., Zhang Y., Yang M., Cao X., Wu J., Yue X. (2016). Effect of Taurine from Bovine Liver on Immune Functions and Antioxidant Capacity in Immunosuppressed Mice. Meat. Res..

[8] Hou J., Jia Y., Yang Z., Li Y., Cheng F., Li D., Ji F. (2013). Effects of Taurine Supplementation on Growth Performance and Antioxidative Capacity of Chinese Soft-Shelled Turtles, *Pelodiscus sinensis*, Fed a Diet of Low Fish Meal Content. J. World Aquacult. Soc..

[9] Qian W., Li M., Yu L., Tian F., Zhao J., Zhai Q. (2023). Effects of Taurine on Gut Microbiota Homeostasis: An Evaluation Based on Two Models of Gut Dysbiosis. Biomedicines.

[10] Forbes Z., Scro A., Patel S., Dourdeville K., Prescott R., Smolowitz R. (2023). Fecal and Cloacal Microbiomes of Cold-Stunned Loggerhead *Caretta caretta*, Kemp’s Ridley *Lepidochelys kempii*, and Green Sea Turtles *Chelonia mydas*. Endanger. Species Res..

[11] Robinson C.J., Bohannan B.J.M., Young V.B. (2010). From Structure to Function: The Ecology of Host-Associated Microbial Communities. Microbiol. Mol. Biol. Rev..

[12] Macke E., Tasiemski A., Massol F., Callens M., Decaestecker E. (2017). Life History and Eco-evolutionary Dynamics in Light of the Gut Microbiota. Oikos.

[13] Li Y., Xu Z., Liu H. (2021). Nutrient-Imbalanced Conditions Shift the Interplay between Zooplankton and Gut Microbiota. BMC Genom..

[14] Costello E.K., Gordon J.I., Secor S.M., Knight R. (2010). Postprandial Remodeling of the Gut Microbiota in *Burmese pythons*. ISME J..

[15] Hong P.-Y., Wheeler E., Cann I.K.O., Mackie R.I. (2011). Phylogenetic Analysis of the Fecal Microbial Community in Herbivorous Land and Marine Iguanas of the Galápagos Islands Using 16S rRNA-Based Pyrosequencing. ISME J..

[16] Turnbaugh P.J., Hamady M., Yatsunenko T., Cantarel B.L., Duncan A., Ley R.E., Sogin M.L., Jones W.J., Roe B.A., Affourtit J.P. (2009). A Core Gut Microbiome in Obese and Lean Twins. Nature.

[17] Ley R.E., Bäckhed F., Turnbaugh P., Lozupone C.A., Knight R.D., Gordon J.I. (2005). Obesity Alters Gut Microbial Ecology. Proc. Natl. Acad. Sci. USA.

[18] Zhu L., Wu Q., Dai J., Zhang S., Wei F. (2011). Evidence of Cellulose Metabolism by the Giant *Panda* Gut Microbiome. Proc. Natl. Acad. Sci. USA.

[19] Zhao C. (2018). The Breeding Status and Prospect of Native Turtles in China. China Fish..

[20] Arena P.C., Warwick C., Steedman C. (2013). Welfare and Environmental Implications of Farmed Sea Turtles. J. Agric. Environ. Ethics.

[21] Pan X., Xian j., Kou H., Wang A., Miao Y. (2014). Effects of Dietary Taurine Supplementation on Ingestion and Growth Performance of *Pelodisous sinensis*. Mod. Agric. Sci. Technol..

[22] Sepulveda J., Moeller A.H. (2020). The Effects of Temperature on Animal Gut Microbiomes. Front. Microbiol..

[23] Rawski M., Mans C., Kierończyk B., Świątkiewicz S., Barc A., Józefiak D. (2018). Freshwater Turtle Nutrition—A Review of Scientific and Practical Knowledge. Ann. Anim. Sci..

[24] Wang X., Shang Y., Wei Q., Wu X., Dou H., Zhang H., Zhou S., Sha W., Sun G., Ma S. (2021). Comparative Analyses of the Gut Microbiome of Two Fox Species, the Red Fox (*Vulpes vulpes*) and Corsac Fox (*Vulpes corsac*), that Occupy Different Ecological Niches. Microb. Ecol..

[25] Guo X.H., Guo Y.L., Liu Y.D., Liu J., Shi W.Q., Dong L., Cai C.B., Cao G.Q., Li B.G., Gao P.F. (2019). Analysis of Colonic Microbiota Characteristics in Piglets at Different Developmental Stages. Acta Vet. Zootech. Sin..

[26] Li C.-J. (2023). Comparative Genomic Analysis and Proposal of *Clostridium yunnanense* sp. Nov., *Clostridium rhizosphaerae* sp. Nov., and *Clostridium paridis* sp. Nov., Three Novel Clostridium Sensu Stricto Endophytes with Diverse Capabilities of Acetic Acid and Ethanol Production. Anaerobe.

[27] Guo P., Zhang K., Ma X., He P. (2020). Clostridium Species as Probiotics: Potentials and Challenges. J. Anim. Sci. Biotechnol..

[28] Bilen M., Mbogning M.D., Cadoret F., Dubourg G., Daoud Z., Fournier P.E., Raoult D. (2017). ‘*Pygmaiobacter massiliensis*’ sp. Nov., a New Bacterium Isolated from the Human Gut of a Pygmy Woman. New Microbes New Infect..

[29] Shakir R. (2021). Brucellosis. J. Neurol. Sci..

[30] Lambert S., Thébault A., Anselme-Martin S., Calenge C., Dunoyer C., Freddi L., Garin-Bastuji B., Guyonnaud B., Hars J., Marchand P. (2023). La brucellose du bouquetin des Alpes: Un exemple de dix années de recherche et d’expertise. Med. Sci..

[31] Khairullah A., Kurniawan S., Puspitasari Y., Aryaloka S., Silaen O., Yanestria S., Widodo A., Moses I., Effendi M., Afnani D. (2024). Brucellosis: Unveiling the Complexities of a Pervasive Zoonotic Disease and Its Global Impacts. Open Vet. J..

[32] Daniel H. (2023). Gut Physiology Meets Microbiome Science. Gut Microbiome.

[33] Kuziel G.A. (2022). The Gut Microbiome. Curr. Biol..

[34] Ahasan M.S., Waltzek T.B., Huerlimann R., Ariel E. (2017). Fecal Bacterial Communities of Wild-Captured and Stranded Green Turtles (*Chelonia mydas*) on the Great Barrier Reef. FEMS Microbiol. Ecol..

[35] Ahasan M.S., Kinobe R., Elliott L., Owens L., Scott J., Picard J., Huerlimann R., Ariel E. (2019). Bacteriophage versus Antibiotic Therapy on Gut Bacterial Communities of Juvenile Green Turtle, *Chelonia mydas*. Environ. Microbiol..

[36] Rafael G.-P., Filipa G.-V., Anne J., Arnold R.-H., Herminio G., Jessica B., Fahcina L., Oluwasina F., Christina M., Sidransky D. (2016). 16S rRNA Amplicon Sequencing Identifies Microbiota Associated with Oral Cancer, Human Papilloma Virus Infection and Surgical Treatment. Oncotarget.

[37] Chen S., Zhou Y., Chen Y., Gu J. (2018). Fastp: An Ultra-Fast All-in-One FASTQ Preprocessor. Bioinformatics.

[38] Magoč T., Salzberg S.L. (2011). FLASH: Fast Length Adjustment of Short Reads to Improve Genome Assemblies. Bioinformatics.

[39] Edgar R.C. (2013). UPARSE: Highly Accurate OTU Sequences from Microbial Amplicon Reads. Nat. Methods.

[40] Wang Q., Garrity G.M., Tiedje J.M., Cole J.R. (2007). Naïve Bayesian Classifier for Rapid Assignment of rRNA Sequences into the New Bacterial Taxonomy. Appl. Environ. Microbiol..

[41] Schloss P.D., Westcott S.L., Ryabin T., Hall J.R., Hartmann M., Hollister E.B., Lesniewski R.A., Oakley B.B., Parks D.H., Robinson C.J. (2009). Introducing Mothur: Open-Source, Platform-Independent, Community-Supported Software for Describing and Comparing Microbial Communities. Appl. Environ. Microbiol..

[42] Ji P., Rhoads W.J., Edwards M.A., Pruden A. (2017). Impact of Water Heater Temperature Setting and Water Use Frequency on the Building Plumbing Microbiome. ISME J..

