# Supplementary material for: Dietary Taurine Regulation of the Intestinal Microbiome in Chinese Stripe-Necked Turtle (Mauremys sinensis)
Source: Int J Mol Sci. 2025 Jan 7;26(2):445. doi: 10.3390/ijms26020445 (PMC11764648; doi:10.3390/ijms26020445)
Supplement: Supplementary file 1 [file ijms-26-00445-s001.zip › ijms-3365100-supplementary.pdf]

Number and length sequences in the samples.

Sample\Info	Seq_num	Base_num	Mean_length	Min_length	Max_length
Control_1	35535	14383605	404.7729	360	430
Control_2	31146	12717939	408.33298	397	430
Control_3	43530	17725730	407.207213	275	492
Control_4	45906	18626849	405.760663	399	445
Control_5	42870	17462877	407.344927	360	430
Control_6	39994	16377288	409.493624	360	430
Low_1	47661	19431820	407.709028	360	430
Low_2	50898	20641241	405.541298	396	430
Low_3	61891	25284151	408.527104	248	431
Low_4	46735	18978131	406.079619	360	430
Low_5	53288	21581617	404.999568	360	430
Low_6	45410	18380225	404.761616	274	430
High_1	44102	17973611	407.546392	393	430
High_2	63641	26662474	418.951211	353	430
High_3	43156	17677090	409.609093	396	444
High_4	46289	18776771	405.642183	360	449
High_5	56146	23055821	410.640491	360	430
High_6	40897	16584719	405.524097	234	430
